# Temperature Effect on Exploitation and Interference Competition among *Microcystis aeruginosa*, *Planktothrix agardhii* and, *Cyclotella meneghiniana*

**DOI:** 10.1155/2015/834197

**Published:** 2015-08-25

**Authors:** Andreia Maria da Anunciação Gomes, Sandra Maria Feliciano de Oliveira e Azevedo, Miquel Lürling

**Affiliations:** ^1^Laboratório de Ecofisiologia e Toxicologia de Cianobactérias, IBCCF, Universidade Federal do Rio de Janeiro, CCS, Bloco G, 21949-900 Rio de Janeiro, RJ, Brazil; ^2^Laboratório de Botânica, Instituto de Recursos Naturais, Universidade Federal de Itajubá, Avenida BPS 1303, Pinheirinho, 37500-903 Itajubá, MG, Brazil; ^3^Aquatic Ecology & Water Quality Management Group, Department of Environmental Sciences, Wageningen University, 6708 PB Wageningen, Netherlands; ^4^Department of Aquatic Ecology, Netherlands Institute of Ecology (NIOO-KNAW), 6708 PB Wageningen, Netherlands

## Abstract

We studied the effect of temperature (18 and 30°C) on growth and on the exploitation and interference competition of three species:* Microcystis aeruginosa* (MIJAC),* Planktothrix agardhii* (PAT), and* Cyclotella meneghiniana* (CCAP). Coculturing the organisms in batch systems allowed for the examination of both competitive interactions, while the interference competition was studied in cross-cultures. The experiments were done during 10–12 days, and samples were taken for chlorophyll-*a* analysis, using PHYTO-PAM. The temperature did not influence exploitation competition between MIJAC and other competitors and it was the best competitor in both temperatures. PAT presented higher growth rates than CCAP in competition at 18 and 30°C. The temperature influenced the interference competition. The growth of MIJAC was favored in strains exudates at 30°C, while CCAP was favored at 18°C, revealing that the optimum growth temperature was important to establish the competitive superiority. Therefore, we can propose two hypotheses: (i) different temperatures may results in production of distinct compounds that influence the competition among phytoplankton species and (ii) the target species may have different vulnerability to these compounds depending on the temperature. At last, we suggest that both the sensitivity and the physiological status of competing species can determine their lasting coexistence.

## 1. Introduction

Species of cyanobacteria are common members of phytoplankton communities in any aquatic environment and some of them can form persistent blooms. These blooms have been associated with anthropogenic eutrophication [1]. To date, there are a several environmental conditions, including nutrient availability and temperature changes, which are being described as important drivers to phytoplankton composition [2]. For example, based on data collected starting in 1996, a shallow tropical eutrophic costal lagoon in Brazil has shown that cyanobacteria can reach up to 90% of the total phytoplankton biomass during some year seasons [3]. However, different species of cyanobacteria, like* Microcystis aeruginosa*,* Planktothrix agardhii*, and* Aphanizomenon* sp., have presented an alternate dominance in this phytoplankton community during high nutrient and elevated temperature periods. Furthermore in lower temperature periods, some diatoms, like* Cyclotella meneghiniana* and* Thalassiosira* sp., could be observed (personal data, unpublished).

Competition is a major regulatory factor in population and community dynamics. However, competition among phytoplanktonic groups is not restricted to physical and chemical resources but it also can include an active process involving release of some organic compounds that directly interfere with competitors, which is in accordance with allelopathy definition described by Rice [4]. The role of exploitation competition and allelopathy (interference competition) in aquatic systems has received increasing attention, especially as a potential means of controlling blooms [5]. The best competitor confers advantage over other phytoplankton species in order to occupy a niche. The mechanism of allelochemical action depends on the interaction between the producer species and the target species. The main mechanisms of action described for phytoplankton are photosynthesis inhibition, enzyme inhibition, cell paralysis, inhibition of nucleic acid synthesis, and reactive oxygen species production [6]. On the other hand, the stimulating effects are nutrients or vitamins releases (previously stored by the producer), bioactive secondary metabolites production, and antibacterial and antifungal production that will benefit other phytoplankton organisms [7–9]. Allelochemical production also depends on physiological conditions, and some physical-chemical factors of the environment can influence the allelopathic mechanism, such as nutrient availability, light intensity, pH, and temperature [9–13].

Temperature is among the major determinants which influences phytoplankton growth rates, nutrient stoichiometry, and spatial and temporal distribution in freshwater systems. Many studies report that lower temperatures favor the growth of diatoms, whereas cyanobacteria growth is favored at higher temperatures, showing that the competitive abilities of these organisms differ depending on environmental conditions [1, 2]. The effect of temperature on the physiology of resource use by phytoplankton, including nutrient uptake [14, 15] and the chemical composition of the cells [16, 17], has been discussed during the last decades. Temperature can directly influence the growth of phytoplankton by altering their metabolic processes and nutrient uptake rates [18–20].

Changes in phytoplankton composition in a eutrophic tropical lagoon, studied by our group during the last two decades, have been associated with temperature changes [3]. In order to better understand this relationship, we chose the most representative species from this system to test the temperature effect on exploitation and interference competition among some phytoplanktonic species. Therefore, we studied the effect of two distinct temperatures (18 and 30°C) on growth and competition of two cyanobacteria (*M. aeruginosa* (MIJAC) and* P. agardhii* (PAT)) and a diatom (*C. meneghiniana* (CCAP)).

## 2. Material and Methods

### 2.1. Algal Species and Culture Conditions

The effects of temperature on growth and competition between* Microcystis aeruginosa* (Kützing) Kützing 1846, strain MIJAC-01,* Planktothrix agardhii* (Gomont) Anagnostidis & Komárek 1988, strain PAT-3, and* Cyclotella meneghiniana* Kützing 1844, strain CCAP1070/5 were studied in batch cultures. The* M. aeruginosa* (MIJAC-01) strain was isolated from a shallow eutrophic lagoon, located in southeastern Brazil (Jacarepaguá Lagoon, 22° 55′ S and 43° 17′ W).* P. agardhii* (PAT-3) was obtained from the Aquatic Ecology and Water Quality Laboratory of Wageningen University and it was isolated from a German water body, and* C. meneghiniana* (CCAP1070/5) was purchased from Culture Collection of Algae and Protozoa (CCAP). All strains were grown in modified WC- (Woods Hole modified CHU10-) mediums [21], at a light intensity of 60 *μ*mol photons m^−2^ s^−1^ using cool white fluorescent light with a regime of 14 h light : 10 h dark. Before the beginning of each experiment, cultures were acclimated to each tested temperature for 10 days. Strains were not axenic, but regular microscopic inspection revealed that biomass of heterotrophic bacteria remained well under 1% of total biovolume.

Mixed cultures of organisms in batch systems allowed for the examination of exploitation and interference competitive interactions, while only interference competition was studied in cross-cultures.

### 2.2. Temperature Effect on Growth: Monocultures Experiments

In order to evaluate the effect of temperature on growth of different strains,* M. aeruginosa* (MIJAC),* P. agardhii* (PAT), and* C. meneghiniana* (CCAP) were cultivated in modified WC medium under 60 *μ*mol photon m^−2^ s^−1^ of light intensity, photoperiod regime of 14 : 10 h (light/dark), and 60 rpm agitation. The experiments were performed in 200 mL Erlenmeyer flasks with a culture medium volume of 100 mL in acclimated incubators at 18, 21, 24, 27, and 30°C. These temperatures were chosen based on minimum and maximum ranges of water temperature averages recorded in aquatic systems from tropical regions. The experiment was run in triplicate, and growth was monitored for 10 days. Chlorophyll-*a* concentration in each flask was measured daily during experimental period using a PHYTO-PAM phytoplankton analyzer (Heinz Walz GmbH, Effeltrich, Germany).

### 2.3. Temperature Effect on Competition: Mixed Cultures Experiments

The effect of temperature on competition among species was evaluated with mixed cultures of* M. aeruginosa* (MIJAC),* P. agardhii* (PAT), and* C. meneghiniana* (CCAP) at two different temperatures (18°C and 30°C). The initial biovolume for each species was 10^7^ *μ*m^3^·mL^−1^. The mixed cultures were (a) MIJAC + PAT, (b) MIJAC + CCAP, (c) PAT + CCAP, and (d) MIJAC + PAT + CCAP. The monocultures (used as a control) and mixed cultures were performed in triplicate for 12 days. The light intensity (60 *μ*mol photons m^−2^ s^−1^), photoperiod (14 h ligth : 10 h dark), and agitation (60 rpm) were controlled. The growth was monitored by chlorophyll-*a* concentrations, which were measured using the PHYTO-PAM phytoplankton analyzer on alternate days. This equipment can easily discriminate the brown, blue, and green pigments from phytoplankton, and it was used to differentiate between the chlorophyll-*a* concentration of diatom and cyanobacteria. To monitor the growth of each cyanobacteria strain in mixed culture, we counted the number of cells of each species within the same sample using a hemocytometer (Fuchs-Rosenthal chamber) and then, to estimate the chlorophyll-*a* concentration from each cyanobacteria strain, we divided the concentration by the number of cells contained in each one. Based on previous experiments, we assumed that MIJAC (*M. aeruginosa*) and PAT (*P. agardhii*) strains produced the same concentration of chlorophyll-*a* per cell.

### 2.4. Temperature Effects on Interference Competition: Cross-Cultures Experiments

The previous mixed culture experiment implies both exploitation competition and interference competition acting simultaneously. In order to distinguish between both types of possible interactions, monocultures of* M. aeruginosa* (MIJAC),* P. agardhii* (PAT), and* C. meneghiniana* (CCAP) were grown in culture filtrates (exudates) of other species, as well as the mixed culture of these three species (MIJAC + PAT + CCAP) at 18°C and 30°C. They were grown in modified WC-medium batch cultures, at the same culture condition described for the mixed cultures experiment. The initial biovolume of each monoculture and each strain of mixed culture was 5.0 × 10^7^ *μ*m^3^·mL^−1^. The final (after 10 days) total biovolume (measured using CASY-counter analyzer) of the three-mixed culture and cell densities of each strain, in mixed culture, are described in Table 1. After 10 days of growth, these cultures were carefully filtered with a 0.2 *μ*m membrane filter (Schleicher & Schell Microscience, Germany) into sterilized filtration systems. Sterilized nutrients were added into exudates at the same concentrations that they are found in the modified WC medium to avoid nutrient limitation; for example, the culture filtrates had an amount of dissolved nitrogen and phosphorus that was not used from the strains after 10 days of cultivation; then, the same nutrient concentration of WC medium was added into these filtrates, representing an overall increase in nutrient concentration. Dissolved nitrogen (nitrite, nitrate, and ammonium) and phosphorus were analyzed in exudate of each monoculture and mixed culture (before the medium reconstruction), following the methodology described in APHA [22]. Dissolved organic and inorganic carbon (DOC and DIC) were also determined in exudates and these values are detailed in Table 2.

**Figure 1:**
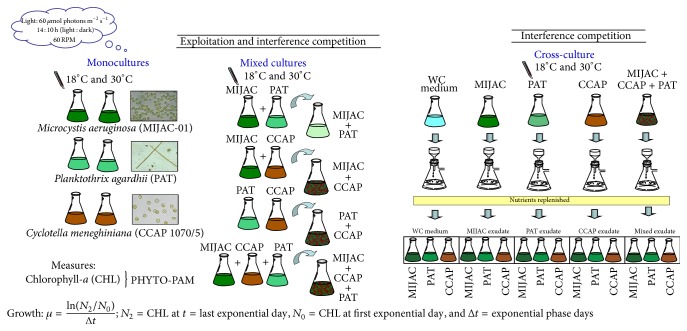
Experimental design of temperature effect on monoculture growth and direct and indirect competition among* Microcystis aeruginosa*,* Planktothrix agardhii*, and* Cyclotella meneghiniana.*

The experimental set was carried out as described below: (a) MIJAC growth on PAT monoculture exudate, (b) MIJAC growth on CCAP monoculture exudate, (c) PAT growth on MIJAC monoculture exudate, (d) PAT growth on CCAP monoculture exudate, (e) CCAP growth on MIJAC monoculture exudate, (f) CCAP growth on PAT monoculture exudate, (g) growth of the three monospecific strains in their own exudates, and (h) each strain growth on MIJAC, PAT, and CCAP mixed culture exudate (the experimental design is in Figure 1). The monoculture of each strain grown in modified WC medium was used as control. The initial biomass of the target strain was 10^7^ *μ*m^3^·mL^−1^. The culture condition was the same one described above. The experiments were performed in triplicate and the growth was monitored during 12 days by analyzing the chlorophyll-*a* concentrations using the PHYTO-PAM phytoplankton analyzer on days 0, 2, 4, 6, 8, 10, and 12.

### 2.5. Data Analysis

Specific growth rates were calculated according to the equation *μ* = ln(*N*_*t*_ − *N*_0_)/Δ_*t*_, where *μ* is the growth rate, *N*_0_ and *N*_*t*_ are chlorophyll-*a* concentrations values at the beginning and the end of the exponential phase, respectively, and Δ_*t*_ is the period, in days, of the exponential phase. Δ_*t*_ was checked by the value of the correlation coefficient (*r* ^2^) greater than 95%.

A one-way analysis of variance (ANOVA) with temperature (18, 21, 24, 27, and 30°C) as fixed factor was performed in order to test whether temperature affects the growth rate of the monocultures (*M. aeruginosa*,* P. agardhii*, and* C. meneghiniana*) and mixed cultures. ANOVA with culture exudates (*M. aeruginosa* exudates (Ma exud.);* P. agardhii* exudates (Pa exud.);* C. meneghiniana* exudates (Cm exud.);* M. aeruginosa* +* P. agardhii* +* C. meneghiniana* exudates (Ma + Pa + Cm exud.) and WC medium) as fixed factor was used for multiple comparison of cross-culture experiments performed in this study. Post hoc analyses were conducted using Tukey's test and *p* value <0.05 was considered statistically significant. All of the statistical analysis was performed using SPSS 17.0 statistical program.

## 3. Results and Discussion

### 3.1. Temperature Effect on Monocultures

**Figure 2:**
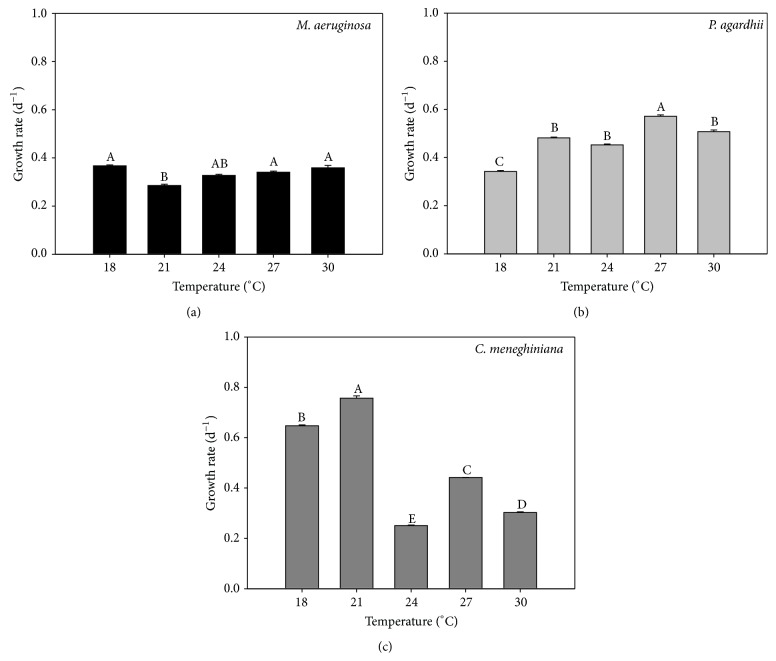
Chlorophyll-a-based growth rate of* M. aeruginosa *(a),* P. agardhii *(b), and* C. meneghiniana *(c) cultured at different temperatures. Different letters represent significant differences at *p* < 0.05.

The reasons for seasonal variability in the phytoplankton composition are poorly understood but may include exploitation and interference competition. Collectively, our results suggest that the temperature can affect the growth, exploitation, and interference competition, but the response is dependent on the species.* M. aeruginosa* was the strongest competitor in all tested conditions. Temperature was not an important factor in* M. aeruginosa* growth in monocultures. Similar growth rates were observed for* M. aeruginosa* grown from 18°C to 30°C (Figure 2(a)). Possibly, even lower temperatures could affect the growth rate of this species, as Soares [23] described for* M. aeruginosa* growing at 12°C. High temperatures also did not increase its growth rate, although some authors have reported that the optimum growth temperature for* M. aeruginosa* is between 25 and 30°C [1, 24, 25]. On the other hand,* P. agardhii* growth was favored at higher temperatures and our results do corroborate those of Lürling et al. [25], showing that the optimum growth temperature for* P. agardhii* is around 27°C (Figure 2(b)). The variation of the* C. meneghiniana* growth rate in response to temperature is in agreement with the observations found for diatom species. Its growth was favored at lower temperatures (18°C and 21°C; Figure 2(c)). When we observe its distribution in water bodies, actually diatoms dominate the phytoplankton community at lower water temperatures [3, 26]. It is also interesting to observe that in these experimental conditions the highest growth rate among three tested strains was registered for* C. meneghiniana*.

### 3.2. Temperature Effect on Competition: Mixed Cultures Experiments

**Figure 3:**
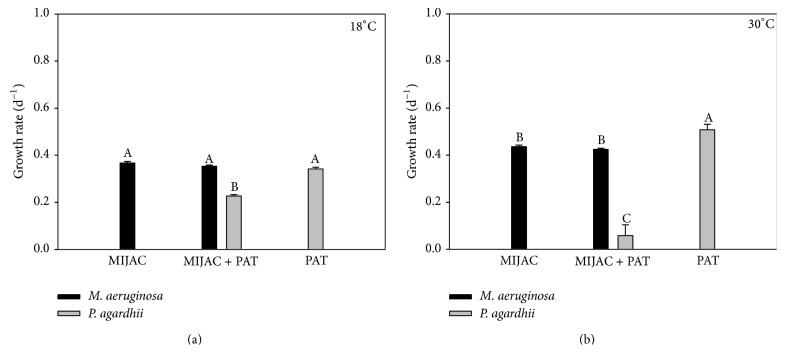
Chlorophyll-*a*-based growth rate of monocultures and mixed cultures of* M. aeruginosa* (MIJAC) and* P. agardhii *(PAT) at two different temperatures (18°C and 30°C). Different letters represent significant differences at *p* < 0.05.

**Figure 4:**
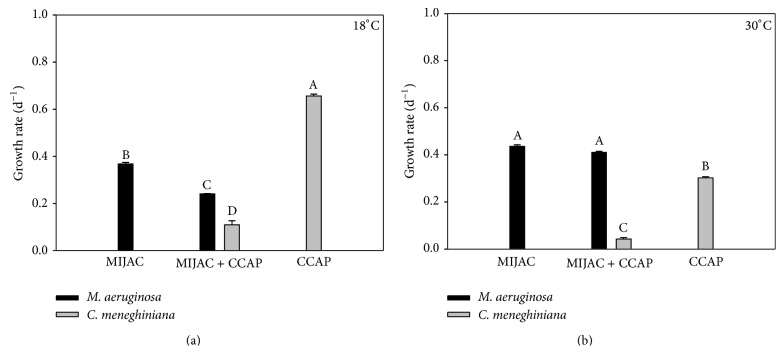
Chlorophyll-*a*-based growth rate of monocultures and mixed cultures of* M. aeruginosa* (MIJAC) and* C. meneghiniana *(CCAP) at two different temperatures (18°C and 30°C). Different letters represent significant differences at *p* < 0.05.

**Figure 5:**
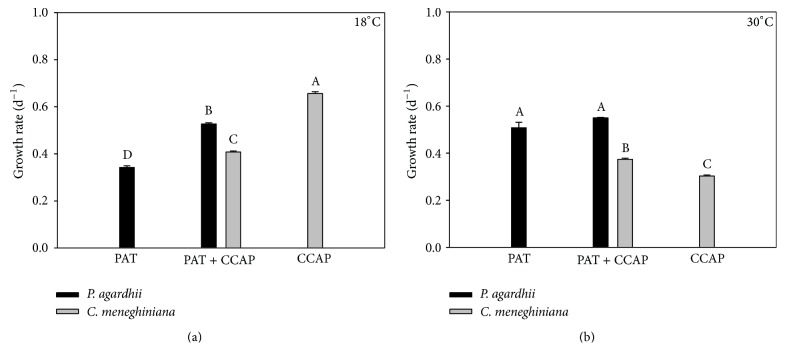
Chlorophyll-*a*-based growth rate of monocultures and mixed cultures of* P. agardhii* (PAT) and* C. meneghiniana *(CCAP) at two different temperatures (18°C and 30°C). Different letters represent significant differences at *p* < 0.05.

**Figure 6:**
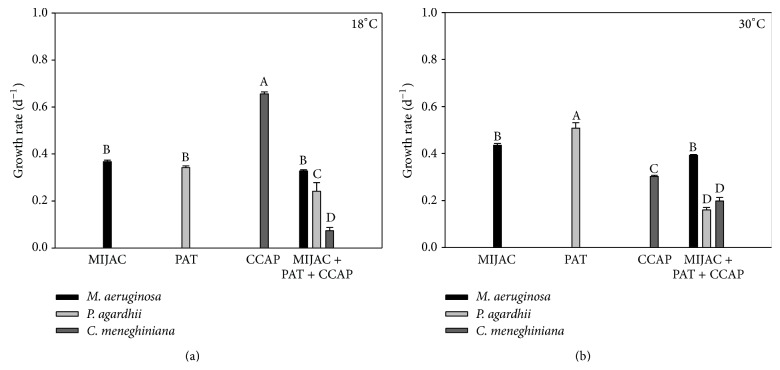
Chlorophyll-*a*-based growth rate of monocultures and mixed cultures of* M. aeruginosa *(MIJAC),* P. agardhii* (PAT), and* C. meneghiniana *(CCAP) at two different temperatures (18°C and 30°C). Different letters represent significant differences at *p* < 0.05.

Temperature also did not have an influence on competition among* M. aeruginosa* and other competitors.* M. aeruginosa* was a stronger exploitation/interference competitor (mixed culture) in both temperatures (Figures 3(a), 3(b), 4(a), and 4(b)). Although* M. aeruginosa* has showed reduced growth rates in the coculture with* C. meneghiniana* at 18°C, compared with the control (monoculture), it still won the competition. Lower temperatures favored the growth of* C. meneghiniana* both in monocultures and in mixed cultures, but its growth rate was still lower than that of* M. aeruginosa* in coculture (Figure 4(a)).* P. agardhii* inhibited the growth of* C. meneghiniana* at 18°C (Figure 5(a)) and stimulated it at 30°C (Figure 5(b)). The presence of* C. meneghiniana* increased the growth rate of* P. agardhii* at 18°C (Figure 5(a)) and none influence at 30°C was observed. In the three-mixed culture,* P. agardhii* and* C. meneghiniana* had their growth rates reduced in both temperatures. On the other hand, the presence of these two competitors did not influence* M. aeruginosa* growth (Figures 6(a) and 6(b)).

The competitive success of* M. aeruginosa* may also be related to its greater ability for nutrients acquisition.* Microcystis* has mean values of half-saturation constant for phosphorus uptake (Ks) of 0.5 *μ*g P L^−1^, while Ks (P) for* C. meneghiniana* is 8 *μ*g P L^−1^ [27]. It means that* M. aeruginosa* has higher growth rate than other strains; in addiction, it has a lower requirement for P, which makes this species an excellent competitor.* P. agardhii* also presented higher growth rate than* C. meneghiniana* at 18 and 30°C in coculture (Figures 5(a) and 5(b)), and its Ks (P) is 1.0 *μ*g P L^−1^. Furthermore,* Planktothrix* has low requirement for light, because it has high phycoerythrin pigment content [27, 28]. The presence of* C. meneghiniana* also stimulated the growth of* P. agardhii* at 18°C.

### 3.3. Temperature Effects on Interference Competition: Cross-Cultures Experiments

**Figure 7:**
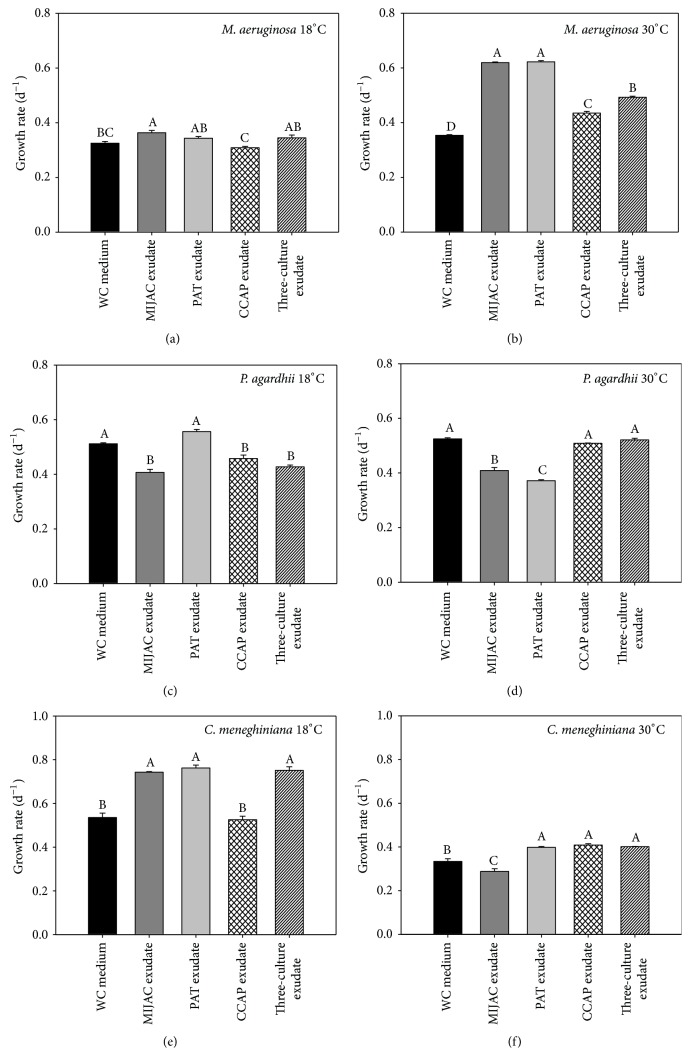
Chlorophyll-*a*-based growth rate of* M. aeruginosa*,* P. agardhii,* and* C. meneghiniana* cross-culture at two different temperatures (18°C and 30°C). Different letters represent significant differences at *p* < 0.05.

Differences in responses of target species suggest that the competitor organisms may produce multiple compounds that vary in their allelopathic potential as a function of species, strains, or even environmental factor [9, 29]. Our study indicated that temperature was also an important factor in the interference competition. The growth of* M. aeruginosa* was favored by all competitor exudates at 30°C (Figures 7(a) and 7(b)), which is frequently described as its optimum growth temperature [25].* C. meneghiniana* was also stimulated by* M. aeruginosa*,* P. agardhii*, and three-culture exudates at its optimum growth temperature (18°C; Figures 7(e) and 7(f)). However,* M. aeruginosa* was not clearly influenced at 18°C and neither* C. meneghiniana* at 30°C. No pattern of response was observed for* P. agardhii* (Figures 7(c) and 7(d)). We cannot determine the exact mechanism responsible for stimulatory effect of competitor exudates on* M. aeruginosa* and* C. meneghiniana*, but it seems that at their optimum growth temperature, these species can make better use of the available resources. The ability of* M. aeruginosa* to use better the available resources should explain its competitive superiority, which is supposed to be able to take up organic matter from the extracellular medium and these compounds favor its development. Many phytoplankton species are capable of using dissolved organic compounds [30, 31]. Cyanobacteria produce many bioactive secondary metabolites, which phytoplankton may utilize for their own metabolism [32]. In the study of Carey and Rengefors [7], the cyanobacterium* Gloeotrichia* stimulated the growth of other phytoplankton, including* Microcystis* and* Cyclotella* species. They suggest that the positive effect may be by released nutrients, such as stored phosphorus and nitrogen. In our study, all species exudates had similar nutrient concentration, not justifying different responses (Table 2). Another possibility suggested was antibacterial and antifungal compounds production by cyanobacteria, which may benefit other phytoplankton species [7, 9].

Allelopathic interaction has also been considered more effective in environmental stress condition [11, 33]. Granéli and Johansson [34] observed an increased allelochemicals production by* Prymnesium parvum* grown under nutrients deficiency. Issa [12] noted that the antibiotic production by cyanobacteria* Oscillatoria angustissima* and* Calothrix parietina* was temperature dependent, but biomass independent. In this study, the temperature stress may have induced* C. meneghiniana* and* P. agardhii* to produce bioactive compounds which improve* M. aeruginosa* growth at 30°C. And* C. meneghiniana* could be favored by temperature stress that* M. aeruginosa* and* P. agardhii* suffered at 18°C.

Our results are in contrast with those showed by Mello et al. [35], and even Sukenik et al. [36] and Vardi et al. [37], where* Microcystis* was inhibited and not stimulated by release of other phytoplankton species. Moreover, in the study of Mello et al. [35], the growth inhibition was observed only in mixed culture exudates, suggesting that the presence of* Microcystis* induced the release of growth inhibitors in* Cylindrospermopsis*. The growth inhibitory effect among* M. aeruginosa*,* P. agardhii*, and* C. meneghiniana* was observed only in coculture assays. It means that cell contact should be important to reveal a growth inhibition between phytoplankton species. At least, it makes sense to avoid the cost of releasing compounds when they are not needed [35] or we can suggest that exploitation competition was the most important interaction able to show growth inhibition in our assays. Otherwise, the three-culture exudates never intensified the positive effect on* M. aeruginosa* at 30°C and neither did* C. meneghiniana* at 18°C.

## 4. Conclusion

Temperature could have influenced qualitatively and/or quantitatively compounds within the exudates, inducing different responses.* M. aeruginosa* exudates at 18°C inhibited* P. agardhii* and its own growth but stimulated* C. meneghiniana*. No pattern of response was observed for* P. agardhii*. Its growth was inhibited by all competitors at 18°C and by* M. aeruginosa* exudates and its own exudates at 30°C. Therefore, we can propose two hypotheses: (i) different temperatures may result in production of distinct compounds that influence the competition among phytoplankton species and (ii) the target species may have different vulnerability to these compounds depending on the temperature. We suggest that both the sensitivity and physiological status of competing species can determine their lasting coexistence.

At last, our results are consistent with observations from shallow tropical eutrophic lagoon in Brazil, where* M. aeruginosa* bloom is affected when the temperature decrease to 18°C, and the bloom releasing may stimulate* C. meneghiniana*. At 30°C,* M. aeruginosa* bloom is stimulated by other phytoplankton species. Although the mechanism evolved in interference and exploitation competition remains to be elucidated, our data suggest that the effect of temperature in both interactions can be considered as explanation for phytoplankton dynamics.

## Figures and Tables

**Table 1:** Total Biovolume (*μ*m^3^ mL^−1^) and Cell density (cell mL^−1^) of each species in mixed-culture among* M. aeruginosa* (MIJAC), *P. agardhii* (PAT) and *C. meneghiniana* (CCAP), after 10 days of growth.

Culture temperature	Total Biovolume (*µ*m^3^ mL^−1^)	Density (cell mL^−1^)		
	MIJAC + PAT + CCAP	MIJAC	PAT	CCAP
18°C	5.8 × 10^8^	2.6 × 10^6^	8.6 × 10^4^	2.4 × 10^4^
30°C	6.1 × 10^8^	3.2 × 10^5^	1.7 × 10^5^	2.0 × 10^4^

**Table 2:** Nutrient concentrations of cells free culture filtrate of *M. aeruginosa* (MIJAC), *P. agardhii *(PAT), *C. meneghiniana *(CCAP) and three culture (MIJAC + PAT + CCAP) at 18°C and 30°C, before and after nutrients replenishment. LOD: level of detection. LOD for NO_3_ + NO_2_: 0.01 mg L^−1^; LOD for PO_4_: 4 *µ*g L^−1^; LOD for NH_4_: 0.02 mg L^−1^.

Culture	TEMP.	NUTRIENTS									
		NO_3_ + NO_2_ mg L^−1^		PO_4_ *µ*g L^−1^		NH_4_ mg L^−1^		DIC mg L^−1^		DOC mg L^−1^	
		Before	After	Before	After	Before	After	Before	After	Before	After
WC^*∗*^		<LOD	16.71	148.83	1220.68	<LOD	<LOD	0.90	4.37	0.79	25.33

MIJAC	18°C	11.62	28.79	1034.81	2553.40	0.08	<LOD	9.53	9.06	35.07	53.78
PAT	18°C	13.25	31.62	922.32	2575.71	<LOD	<LOD	7.99	8.32	33.80	53.74
CCAP	18°C	14.95	35.18	660.75	2436.57	<LOD	<LOD	6.11	10.32	32.57	54.10
MIJAC + PAT + CCAP	18°C	11.96	31.73	707.83	2383.43	<LOD	<LOD	7.87	11.32	40.12	60.47

MIJAC	30°C	9.62	27.87	1027.78	1860.03	<LOD	<LOD	9.84	13.16	36.41	53.89
PAT	30°C	7.14	27.35	747.02	1809.72	<LOD	<LOD	14.80	16.85	35.20	52.66
CCAP	30°C	14.96	33.34	680.70	1620.79	<LOD	<LOD	8.86	9.18	31.48	50.76
MIJAC + PAT + CCAP	30°C	3.99	22.57	578.25	2172.73	<LOD	<LOD	14.85	14.98	35.27	53.42

^*∗*^WC medium, before—nutrients concentration in deionized water before medium preparation; after—deionized water after nutrients replenishment.
